# Mapping the regulatory landscape of AI in healthcare in Africa

**DOI:** 10.3389/fphar.2023.1214422

**Published:** 2023-08-24

**Authors:** Beverley Alice Townsend, Irvine Sihlahla, Meshandren Naidoo, Shiniel Naidoo, Dusty-Lee Donnelly, Donrich Willem Thaldar

**Affiliations:** ^1^ York Law School, University of York, York, United Kingdom; ^2^ School of Law, University of KwaZulu-Natal, Durban, South Africa

**Keywords:** artificial intelligence, AI, Africa, regulation, landscape, healthcare

## Abstract

**Introduction:** Artificial intelligence (AI)-enhanced technology has seen unprecedented expansion in the recent past. This growth brings with it huge opportunities for the positive transformation of the economy, business, healthcare, and society. However, a critical question is whether, and to what extent, regulatory measures and mechanisms have been implemented to safeguard its design, development, and deployment. This paper offers a scoping exercise that maps the regulatory landscape of AI in healthcare (including health research) in certain African countries.

**Methods:** This research is conducted across 12 African countries: Botswana, Cameroon, The Gambia, Ghana, Kenya, Malawi, Nigeria, Rwanda, South Africa, Tanzania, Uganda, and Zimbabwe. As limited specific AI legislation is found in these African countries, and because AI is informed by ancillary regulatory frameworks, we include data protection, digital health, consumer protection, and intellectual property in our research. A scoping review method was applied with a manual search of digital libraries with search terms customised for each repository consisting of core search terms for the various topics, including, among others, “law,” “regulation,” “artificial intelligence,” “data protection,” “intellectual property,” and “digital health”.

**Results and discussion:** Analysis of the data demonstrated that while in the African countries under investigation there is no sui generis AI regulation, recent developments were found in areas that inform AI adoption, including in digital health, data protection, consumer protection, and intellectual property. Our findings highlight the fragmentation of the African AI regulatory landscape and illustrate the importance of continued AI regulatory development to ensure that Africa is well positioned for future AI adoption in health.

## Introduction

Artificial intelligence (AI) is a pivotal player in the emergence of the Fourth Industrial Revolution (‘4IR’). Although no harmonised definition of AI exists, we take the broadly functionalist perspective that AI is to enable a machine or a mechanical device to function or behave in a manner that would be called intelligent were a human to behave in that manner (McCarthy et al., 2006). AI-enhanced technologies have recently expanded in scale, scope, and complexity, including a diverse range of applications globally (Aitken et al., 2022). One sector where AI holds much promise is in its ability to revolutionise and drive healthcare. AI-based technology deployment is hugely advantageous in enhancing connectivity, facilitating the flow of health information, and in providing healthcare services and delivery. The provision of healthcare in Africa faces many challenges, including a shortage of healthcare resources, an increased burden of disease, a large proportion of the population living in rural areas, and a lack of education and primary healthcare–to name a few. Significant advantage may be harnessed by AI application: *inter alia*, in extending healthcare access, by contributing to early disease detection and prevention, supporting diagnostics and drug development, in disease surveillance and tracking, in public health monitoring, healthcare management and clinical decision-making, and in health research more generally (Topol, 2019).

While the disrupting power of such technologies brings with it unprecedented opportunities for the transformation of society and healthcare in particular, there are also concerns about the way in which AI is designed, developed, and deployed. These concerns range from issues concerning data quality and privacy to explainability and transparency of the algorithms, and issues of social and distributive justice (Fjeld et al., 2020). An analysis of current ethical AI guidelines found that while there was convergence of the normative themes (or principles) of transparency, justice, fairness, non-maleficence, and responsibility across many ethical frameworks, principles such as privacy, solidarity, human dignity, and sustainability were underrepresented (Jobin et al., 2019; World Health Organisation 1A, 2021). Notwithstanding the prevalence of ethical instruments–many of which find application in Africa–a critical question is whether AI in Africa is regulated. By regulation, we mean any form of ‘hard’ law–that is, policies that one can enforce in a court of law. While there is much recent development and debate about the regulation of AI in the Global North, far less attention has been directed toward, and indeed little is known about, the AI regulatory position in the Global South and in Africa, in particular (De Almeida et al., 2021; Schmitt, 2022).

### Related work

Research studies have been conducted on the mapping of global AI ethics guidelines, on the ethical challenges presented by AI-driven technologies in healthcare, and on emergent ethical and rights-based approaches to values and principles for AI adoption and global AI governance. However, limited, if any, research has been done on ascertaining the current AI regulatory landscape in the Global South, and in Africa, in particular (Wang and Siau, 2018; Jobin et al., 2019; Fjeld et al., 2020; Gerke et al., 2020). Radu, for example, has conducted a qualitative comparison of the national strategies of 12 countries: Canada, China, France, Finland, Germany, Japan, Singapore, South Korea, Sweden, the United Arab Emirates, the United Kingdom, and the United States (Radu, 2021), Butcher and Beridze provided a synopsis of current AI governance activities globally (Butcher and Beridze, 2019), Larsson analysed the use of ethics guidelines as a governance tool in the development and use of AI with a focus on the Ethics Guidelines for Trustworthy AI published by the EU Commission’s High-Level Expert Group on Artificial Intelligence (Larsson, 2020), and Cheng and Zeng reported on the global AI governance initiatives and China’s ambition to play a leadership role in nascent global AI governance regimes (Cheng and Zeng, 2022). Concerning Africa, comparatively little research has been done. Brand has reviewed recent international developments and has recounted the insufficiency of only implementing a South African national legal framework–arguing in favour of the introduction of practical instruments of governance, such as an algorithmic impact assessment for measuring and mitigating risk and harm (Brand, 2022). In a not dissimilar vein, Abe and Eurallyah have explored the implications for human rights infringements, and Gwagwa et al. explored the core benefits and challenges of AI adoption in Africa (Gwagwa et al., 2020; Abe and Eurallyah, 2021).

### Aim and scope

This article aims to complement current literature by mapping the regulatory landscape of AI in the health context in Africa, with reference to 12 selected countries. By mapping the regulatory landscape, we mean conducting a scoping review of the most relevant regulatory instruments–that is, those we identify and provide references to as regulatory instruments. We consider *AI in healthcare* broadly, including the development of AI-enhanced technology for use in healthcare practice and in health research. As Africa is the second largest and second most populous continent in the world and as it comprises six per cent of the earth’s total surface area, we caution against viewing such an extensive and diverse region with such heterogeneous populations as one amassed, singularly constructed entity. Africa is a vast continent consisting of 54 sovereign states recognised by the United Nations. In this contribution, when the words “Africa,” “African” or “African continent” are used they are intended to describe in particular the 12 African countries under investigation in our research (and not necessarily all 54 African states or countries)—unless the context dictates otherwise. We have thus restricted the scope of our article, for purposes of practicality, to a selection of 12 African countries: Botswana, Cameroon, The Gambia, Ghana, Kenya, Malawi, Nigeria, Rwanda, South Africa, Tanzania, Uganda, and Zimbabwe. These countries are 1) those English-speaking African countries which 2) hosted research projects as part of the H3Africa programme. The 12 selected countries are not representative of Africa generally, but rather represent a selection of African countries that 1) have regulatory instruments in a language that is understandable to us, the investigators, and 2) which can host health research activity that is relevant from a broader international perspective. These English-speaking African countries were selected for previously hosting Human Heredity and Health in Africa (H3Africa) consortium projects and have been included in the Data Science for Health Discovery and Innovation in Africa (DS-I Africa) Law project, which is funded by the National Institutes of Health (NIH).

### Methodology

First, we investigate whether the selected countries have *sui generis* AI regulatory instruments. Next, we identify regulatory instruments in areas of the law that we suggest are most proximate and relevant to AI in health: digital health law, data protection law, consumer protection law, and intellectual property law. Lastly, we investigate the regulatory authorities in these areas of law, as they can often create regulatory instruments in a dynamic fashion in anticipation of (or in response to) technological developments.

Our investigation follows the style of scoping reviews described in Munn et al. (2018) and Peters et al. (2015). This approach was chosen because of the lack of synthesised comprehensive databases and systematic reviews on the topic (Sucharew and Macaluso, 2019). A scoping review is therefore particularly appropriate to achieve this study’s objective of mapping a wide body of regulatory instruments that may affect the emergent legal regulation of AI in healthcare (Munn et al., 2018).

To ensure a comprehensive and systematic search process, searches of various websites as described in Supplementary Annex II, including the Afriwise (Afriwise, 2022) portal and official government websites, were conducted. Where a keyword search was allowed on the website, we used an array of relevant search terms. If not, the sites were manually searched. Supplementary Annex II at Supplementary Tables S1, S2 set out a comprehensive list of the websites searched and the search terms used. The search protocol was developed and pilot-tested initially in South Africa and Kenya, and then applied to other jurisdictions. Each thematic area was surveyed individually, with 97 country-specific databases examined for AI and data protection, 54 for digital health, 11 for consumer protection and ICT law and 21 for intellectual property. In addition, 22 databases were reviewed for information relating to all countries; these include regional/sub-regional organisations and general legal research websites.

From the search results, the researchers downloaded digital copies of all documents relevant to one or more of the study’s five themes. The criteria for inclusion in the scoping review were that the document be one of the following types: 1) national statute currently in force; 2) gazetted regulations; 3) draft Bill, 4) published government policy or strategy document; 5) ethics code/guideline/policy by health sector regulatory body or international or regional legislative, regulatory or policy instrument; and 6) applicable in one or more of the 12 jurisdictions. The study excluded private sector documents, documents not publicly available on the internet, and draft documents under discussion. The extracted documents were saved in a shared Google drive folder, and were classified in sub-folders by country and thematic area. Duplicates and documents replaced/repealed by a more recent document were then manually removed. A total of 118 documents (listed in Supplementary Annex I Supplementary Tables S1–S5) were then legally analysed by the researchers.

### Limitations

While a full and comprehensive account of the regulatory position in the relevant areas of the law is offered, we do not claim to have captured every provision that may find relevance to AI in health or in health research. The consequences of AI adoption are far-reaching, touching many areas of the law. We have therefore narrowed our enquiry to the areas of the law that are most relevant. It was also not our intention to capture the numerous and varied ethics instruments and other non-regulatory governance measures that may find application to AI in healthcare.

### Analysis

#### Sui generis AI regulation

There are no *sui generis* AI regulatory instruments at a regional African level or in any of the 12 African countries under investigation. However, as AI regulation is more than *sui generis* regulation, certain aspects of the development and deployment of AI are informed by either issue-specific legislation (such as healthcare laws) or sector-specific legislation (such as data protection laws). Our analysis categorises the regulations informing AI adoption in our study along four more generalisable themes: digital health law, data protection law, consumer protection law, and intellectual property law.

#### Digital health law

Regulatory frameworks form part of a tool to assess the maturity of AI within health. Thus, the absence of clear AI regulatory guidelines and policies may, in certain instances, impede the uptake of AI in the healthcare sector (Broadband Commission and Working Group on Digital and AI in Health, 2020). Although no *sui generis* AI regulation exists in the countries under investigation, healthcare is not unregulated. However, integrating AI into the existing healthcare and health research systems can present challenges. If there are regulatory voids, guidance is, to some extent, sought from existing national health statutes, digital health policy documents, professional codes of conduct, and healthcare and health research guidelines.

The World Health Organisation has implemented an integrated African Health Observatory initiative, together with National Health Observatories aimed at providing an informative digital health platform (World Health Organisation, 2022a). The African Union through its auspice, the African Medical Devices Forum (New Partnership for Africa’s Development, 2022), has yet to provide regulatory guidance for the use of AI in clinical healthcare and research. At a subregional level, Kenya and Uganda belong to the IGAD (Intergovernmental Authority on Development) group which has developed a policy and implementation plan on health data sharing (Intergovernmental Authority on Development, 2021a; Intergovernmental Authority on Development, 2022b) efforts. These aspire to integrate cross-border health data sharing that in turn facilitates AI development and healthcare in Africa.

An analysis of statutes governing medical devices in the selected African countries shows that no single piece of legislation explicitly mentions AI or algorithms within the definition of a medical device. Furthermore, when compared to the definition of AI provided by the OECD, the definition of software included in current medical device regulations does not specifically and adequately address novel features of AI software. From the scrutiny of the provisions, ‘software’ is included in the definition of medical devices in three jurisdictions (South Africa, Kenya, and Uganda) (Matovu, 2018). This is broadly construed to include software as a medical device (SaMD) (Townsend, 2020). However, these regulatory frameworks do not sufficiently provide AI system risk classifications (critical, serious, or non-serious categories); oversight mechanisms informing the total product life cycle of SaMDs (including pre-market development, post-market management, change management, and ongoing monitoring); guidance on the analytical and clinical validation of the SaMDs; direction on the testing, training, and validation of the datasets; and verification of the veracity and accuracy of the machine learning or other algorithms that underpin the SaMDs.

Reported cases demonstrate that AI has been instantiated in clinical practice in certain countries under investigation. For instance, South Africa used an AI-driven chest x-ray diagnosis application during the COVID pandemic (Staff Reporter IOL, 2020; Philips Foundation team, 2021). Digital health services for medical advice, appointment booking and the delivery of prescriptions to patients through mobile applications, and AI-powered triage systems have been launched in both Rwanda and Tanzania (Babyl, 2022; Elsa Health, 2022). However, the regulation of SaMDs by the medical health regulators is largely undeveloped, with most countries lacking frameworks providing guidance to digital healthcare modalities and applications. These policies are set out in Supplementary Annex I, Supplementary Table S1.

Most countries under study have standalone digital health policies in place, except for Rwanda and The Gambia where such policies are embedded in the broader healthcare policy. Kenya is the only country that has a standalone E-health Bill that regulates digital health. The implementation and monitoring processes of digital health are, however, sporadic and partly a result of a lack of infrastructure and resources (Akanbi et al., 2012; World Health Organisation, 2022b; Butcher et al., 2021; Karamagi et al., 2022; Odenkuhle et al., 2017; Owoyemi et al., 2020). Countries under study that have developed digital health policies have not, however, established professional guidelines for health practitioners, except for South Africa, Kenya, and Zimbabwe (EXCOM, 2014; Ministry of Health, 2017; Health Professions Council of South Africa, 2022). All countries under study have regulations that provide for informed consent, guide health personnel, and stipulate that medical professionals should be registered. In addition, Kenya’s policy allows informed consent to be obtained electronically in line with the data protection law (Onetrust DataGuidance Regulatory Research Software, 2021; Kenya Government, 2023). Informed consent is of particular importance in health research as it safeguards the autonomy and dignity of research participants. Kenya’s and Ghana’s policies allow e-dispensing and e-prescriptions (The Pharmacy Council, Ghana, 2022; Kenya Government, 2023). Only four countries have professional guidelines and policies on telemedicine, namely, South Africa, Kenya, Zimbabwe, and Ghana. For the remainder of the countries, the position on electronic consent, e-dispensing, and e-prescriptions is unclear. Most countries under study do not have guidance on telemedicine which *inter alia* affects its development, adoption, and use in health research. With the absence of telemedicine and digital health guidance, and specific e-health strategies, the call for reform is supported, for example, in South Africa by Townsend et al. and in Botswana by Ncube et al. (Townsend et al., 2019; Ncube et al., 2022). The lack of development, use, and adoption of telemedicine could be related to *inter alia* resource constraints, and to ethical and legal barriers. Lack of adequate healthcare regulations or policies has been noted as a barrier to the adoption of telemedicine in Africa, with Dodoo et al. recommending that governments adopt a comprehensive e-policy framework including the establishment of strict protocols to monitor and evaluate telemedicine practices (Dodoo et al., 2021). These barriers will similarly stand to affect AI in health research.

However, more research is needed as telemedicine solutions are increasingly leveraging AI, as well as new modalities of delivering healthcare services in under-resourced areas, such as Chatbots and mobile applications, to assist community health workers. Regulation that is outdated and not context-specific and culturally appropriate can thus also act as a barrier to digital technology adoption and innovation. In South Africa, for example, there has been a low uptake of telemedicine by healthcare practitioners (Dodoo et al., 2021) and Donnelly has criticised the overly restrictive South African telemedicine guidelines as potentially stifling lawful and ethical development of AI in healthcare (Donnelly, 2022).

The development and adoption of AI in healthcare relies heavily on availability and access to high-quality clinical health data gained from digital health and health research (European Commission, 2022). Therefore, regulatory frameworks associated with the management of digital health data and health research are a foundational element for further development of AI technologies in healthcare. Most countries in the cohort study have legislation on digital health, and regulations that direct the professional conduct of health personnel in clinical and health research are set out in Supplementary Annex I, at Supplementary Table S2. These regulations determine the collection, storage, curation, management, and analysis of digital health data in research, which is vital for AI development and adoption.

In sum, the regulation of AI adoption in healthcare in the countries under study is undeveloped. None of the studied countries have adopted a proactive approach to the development of legislation governing AI in healthcare. The immaturity of AI in healthcare regulatory systems is exacerbated by further impediments including the lack of financial resources, diminished computing resources and structural infrastructure, and inadequate technical expertise. Unfortunately, these factors stand to delay the implementation of digital health in low- to middle-income countries–including the countries under study (World Health Organisation 1B, 2022). Professional guidelines, informed consent provisions, and healthcare and health research regulations provide some guidance and inform AI use in health contexts.

#### Data protection law

There is a close link between data and AI. AI systems rely on vast quantities of accurate, complete, representative, and quality datasets to train, test, and validate the system. Data that is typically personal - and sometimes sensitive or special category data - is typically ‘research’ data. AI systems also collect, generate, process, and share data–often on a large scale. Good AI regulation is thus intrinsically shaped by good data regulation. The increasing use and processing of such datasets informs many possible privacy challenges, including issues associated with collection, standardisation, anonymity, transparency, data ownership, and the changing conceptions of informed consent.

AI-enhanced technologies pose risks to data privacy in two ways. First, in the unlawful collection, use, and sharing of a person’s personal data, and second in not providing persons with access, control, and autonomy over their data and data use. Legal tensions focus on the increasing requirement to access curated quality datasets and the inherent sensitivity of data, in particular personal information and also the implicit vulnerability to its unethical or unlawful source, use, and disclosure. The use and processing of personal data, and in particular sensitive health data and electronic health records, are well described, as are securing and protecting large-scale data sets against unauthorised collection, access, processing, storage, and distribution (Goodman, 2016; Bari and O’ Neill, 2019; Xafis et al., 2019; Townsend and Thaldar, 2020).

Regional developments in Africa have primarily been instantiated through the African Union Convention on Cyber Security and Personal Data Protection, which was adopted in June 2014, and which introduced substantive claims to information privacy in Africa (African Union, 2014).

The AU Convention sought to harmonise African cyber legislation and to elevate the rhetoric of ‘protection of personal privacy’ to an international level. Moreover, it establishes a normative framework consistent with the African legal, cultural, economic, and social environment, and seeks to balance the use of information and communication technologies with the protection of the privacy of individuals, while guaranteeing the free flow of information across borders. The AU Convention enjoins state parties to establish legal and institutional frameworks for data protection and cybersecurity, encompassing three central issues: electronic transactions, personal data protection, and cybercrimes (African Union, 2014). The AU Convention requires 15 ratifications to enter into force. Recently, on 9 May 2023, it indeed reached 15 ratifications, and is therefore now in force (African Union, 2023).

A further development leading to data protection integration, strengthening collaboration in Africa, and facilitating cross-border data transfers occurred in February 2022 with the endorsement of the AU Data Policy Framework (African Union, 2022). This Framework encourages greater collaboration between AU member states and a coordinated, comprehensive, and harmonised approach to data governance.

In addition, subregional frameworks and agreements as created by the Economic Community of West African States (ECOWAS), the East African Community (EAC), the Economic Community of Central African States (ECCAS/CEMAC), the Intergovernmental Authority on Development (IGAD), and the Southern African Development Community (SADC), have contributed to the protection of the right to privacy and to promoting cyber security and fightingcybercrime (East African Community, 2011; Southern African Development Community, 2013a; Southern African Development Community, 2013b; Southern African Development Community, 2013c; East African Community, 2019; Intergovernmental Authority on Development, 2021a; Intergovernmental Authority on Development, 2021b; Economic Community of Central African States, 2021; Economic Community of West African States, 2021; Intergovernmental Authority on Development, 2022a; Intergovernmental Authority on Development, 2022b).

If Africa once lagged in the development of data protection laws, it has recently remedied this position. Until recently, few, if any, data protection policies had been developed in Africa (Van Gyseghem, 2012; Makulilo, 2015). This, however, has changed significantly. In 2021, of the 145 countries globally with data protection laws, 32 were in Africa with Africa the region of fastest data-protection law expansion (Greenleaf, 2021). The most recent African enactments are Tanzania, Egypt, Uganda, Togo, Nigeria, Kenya, Congo-Brazzaville, Botswana, and Zimbabwe (Tanzania; 2022; ILO, 2020; Uganda, 2019; Togo, 2019; Nigeria, 2019; Kenya, 2023; Congo-Brazzaville, 2019; Botswana, 2018; Zimbabwe, 2021; Wilkinson and Ooijevaar, 2020). Of the 12 countries we investigated, nine had specific data protection laws enacted. Botswana has the Data Protection Act No 32 of 2018 which came into force on 15 October 2021 (with the grace period of 1 year for implementation delayed beyond 15 October 2022). It establishes an Information and Data Protection Commission, yet to be set up, which is mandated to do all things necessary to protect the rights of individuals regarding their personal data and to ensure the effective application of the Botswana Data Protection Act. Both Kenya–one of the few countries whose law contains a specific Privacy-by-Design provision–and Ghana have data protection legislation. In Nigeria, data protection is provided by the Nigerian Data Protection Regulation of 2019, which is subsidiary legislation issued pursuant to the National Information Technology Development Agency Act of 2007. Moreover, the Data Protection Bill, 2020 (anticipated to be passed in 2023) seeks to provide an efficient regulatory framework for the protection of personal data and to regulate the processing of information.

Data protection in Rwanda is governed by law No 058/2021 of 2021 relating to the protection of personal data and privacy. Interestingly, Rwandan law contains a provision in Article 19 giving the data subject the right to request a data controller or data processor to stop processing their personal data which ‘causes or is likely to cause loss, sadness or anxiety to the data subject’ and a provision in Article 25 permitting a data subject to designate an heir to their personal data. In South Africa, data is protected by the Protection of Personal Information Act No 4 of 2013, which came into effect on 1 July 2020, Uganda by the Data Protection and Privacy Act of 2019, and Zimbabwe by the Data Protection Act No 5 of 2021. Tanzania enacted its first Personal Data Protection Law in late 2022, in terms of which provision is made for conducting transfer impact assessments and the stipulation that data collectors submit their privacy policies to the Tanzanian Data Protection Commission for approval.

Although not all countries have specific data protection legislation in place, all countries under investigation have data or privacy protection in some form or another, often embedded in other legislation. Cameroon, for example, has no specific law relating to data protection, although a degree of protection is provided by law No 2010/012 of 21 December 2010 Relating to Cyber security and Cyber criminality in Cameroon, by Law No 2006/018 of 29 December 2006 to Regulate Advertising in Cameroon, and by Law No 2010/013 of 21 December 2010 Regulating Electronic Communications in Cameroon. Moreover, the Constitution of the Republic of Cameroon provides for the privacy of all correspondence and Decree No 2013/0399/PM of 27 February 2013 for modalities of the consumers’ protection in the electronic communication sector states that “consumers in the electronic communication sector have the right to privacy … in the consumption of technologies, goods and services in the electronic communication sector.” Cameroon has ratified certain instruments that protect privacy, including the sub-regional CEMAC Directive No 07/08-UEAC-133-CM-18.

In The Gambia, certain data protection and privacy rules relating primarily to information and communications service providers are provided for in their Information and Communications Act, 2009 and the 2019 Data Protection and Privacy Policy sets out the legal framework for data protection and privacy. Although Malawi does not have any specific data protection laws, a Data Protection Bill, 2021, has been drafted. It promotes data security and provides for data protection and related matters, while the Electronic Transactions and Cyber Security Act 33 of 2016 contains data protection-related provisions. We have included a comprehensive list of data protection laws in Supplementary Annex I, at Supplementary Table S3.

#### Consumer protection law

The debate about AI has focused on data protection requirements and soft law ethics instruments. While general AI regulation remains necessary, it is also vital to address the use of and relationship between AI software as goods that can be sold and the patient as a consumer in respect of the AI product or a healthcare service provided using the AI. Traditional fault-based liability regimes are difficult to implement in relation to harm caused by AI technologies as healthcare practitioners are required to foresee an error and take reasonable steps to meet the required standard of care (Donnelly, 2022; Naidoo et al., 2022). In other words, the law regards a doctor as negligent when they fail to act as a reasonable practitioner would have done in that branch of the profession. Considering the inherent opacity of the complex algorithms that power AI, it is highly unlikely that a doctor could reasonably be expected to anticipate errors that may not even be apparent to the AI developers. Imposing strict liability for harm caused by AI technologies has been extensively explored throughout the literature. However, it may be prudent to first investigate present means of imposing liability before we consider the development of new law/regulation. Many suggest that AI applications may necessitate a more sophisticated product liability regime (Chagal-Feferkorn, 2019), in order to address novel user safety risks found in such systems. The targeted jurisdictions have yet to address this matter and product liability for harm caused by AI is likely to be attributed according to the current consumer protection regime.

All 12 countries provide for consumer protection in relation to the sale of goods. Botswana, Cameroon, The Gambia, Kenya, Malawi, South Africa and Zimbabwe have enacted standalone statutes regulating consumer protection. The position is different elsewhere, where it is regulated alongside (Nigeria and Rwanda) or embedded in (Tanzania) fair competition legislation. While both Ghana (Nkansah, 2015) and Uganda (Zeija, 2018) currently have fragmented frameworks for consumer protection, they too have legislation regulating the sale of goods. The consumer protection legislation that does exist in these jurisdictions is set out in Supplementary Annex I at Supplementary Table S4.

Eleven out of the twelve countries provide for strict product liability of harmful or defective goods in their consumer protection regimes. This means that anyone in the supply chain for the AI product (the goods) can be held strictly liable for harm to the patient (the consumer) if the product does not perform safely or as intended. It is not necessary to prove that the harm arose from any negligence (fault) on the part of the developer or the doctor. Cameroon deviates from this general trend, as the imposition of product liability is negligence-based, that is, a determination of fault is necessary to impose liability (Galega, 2018).

Within current legislation, liability may be wholly or partly imposed on a number of different parties in the distribution chain, such as: the supplier, producer, manufacturer, importer, distributor, trader, seller, retailer, or provider of services (The Gambia, Malawi, and Nigeria). In South Africa, for example, the term supplier is wide enough to include the developer of the AI product and the healthcare establishment or practitioner providing a service using the AI product. Where health researchers intend to commercialise an AI product that they have developed, they too would need to be aware of the legal obligations imposed by consumer protection legislation. In addition, Rwanda’s legislation contains a unique provision in terms of which strict product liability for unsafe or defective goods supplied by an enterprise is imposed upon the regulatory body that approved the product for sale.

A consideration of what types or aspects of technology may be included in the definition of goods is necessary. This becomes especially relevant to AI, given the recent CJEU finding that where the supply of software by electronic means is accompanied by a grant of perpetual licence, this will constitute the sale of goods (The Software Incubator Ltd, 2021). However, only Uganda, South Africa and Zimbabwe explicitly include software in the definition of goods. In seven other countries, software could be included by implication, as the term goods is either undefined (Cameroon, The Gambia, Kenya), or the nature of the goods covered is unspecified–but arguably wide enough–to include software. For example, Botswana defines *commodity* to include corporeal or incorporeal property; Ghana defines goods as ‘movable property of every description’; while in Nigeria and Tanzania, goods are enumerated as–but not limited to–tangible goods. However, in Malawi, software is excluded because the Act applies to tangible goods only.

Definitions of what constitutes a consumer also vary. Seven countries–Botswana, Cameroon, Malawi, Nigeria, Tanzania, Uganda, and Zimbabwe–provide for the explicit exclusion of persons who purchase goods and services for the purpose of reuse in production and manufacture of any other goods or services for sale, and in Rwanda the Act applies only to goods ordinarily acquired for personal and domestic use. This is particularly noteworthy, given that statistically-based machine learning models used in the healthcare context will invariably be acquired for reuse in the production/manufacturing of other goods (e.g., drug discovery) and services (e.g., disease prediction, patient diagnoses, population health monitoring). Thus, those acquiring data-driven AI technologies for the purposes of health research or use in healthcare practice–where the objective is the sale of a good/service–are not themselves defined as consumers and are thus unlikely to find much protection under consumer legislation. In ensuring compliance with legislation, eight countries–Cameroon, The Gambia, Malawi, Nigeria, Rwanda, South Africa, Tanzania, and Zimbabwe–allow the relevant consumer protection authority to issue a recall on any goods considered a risk to the public or harmful to human or public health. The Gambia and Tanzania differ in that the supplier or relevant party of the distribution chain is responsible to recall harmful or defective goods. Furthermore, both The Gambia and Malawi provide for an additional safeguard against harmful technology, goods, and services. Here producers or suppliers are intended to attach easily noticeable warnings to products considered harmful or hazardous to human health with the aim that use take place under the strongest possible safety conditions.

In addition, electronic communications and transactions and the protection of e-consumers are regulated in a number of jurisdictions in other legislation. These statutes, which do not refer in specific terms to AI, also do not contain any provisions that could clarify the attribution of liability or address many of the other significant consumer protection concerns that arise from the use of AI in healthcare. In addition, some jurisdictions have laws regulating cybercrimes, content control measures and service provider liability. These safeguards also do not directly address the issue of providing civil redress to individual consumers harmed by an AI application in the healthcare setting.

#### Intellectual property law

Before one can engage with research, one must first understand the regulatory environment. Importantly, this includes the schemes of protection for any fruits of research. This would be intellectual property. In this section we outline the mechanisms and bodies which are relevant in obtaining such protection. Multiple layers of intellectual property (‘IP’) protection can apply to a single AI product or process. For this research study we focused on only two IP rights: patents and copyright. These IP rights inform data flow, affect AI research and development, and are critical for AI innovation. Patents generally apply to product inventions (such as AI technologies embedded within products, for example, smartwatches). Copyright applies to literary works, which includes the datasets used to test, train, and validate AI systems. Regional IP frameworks were identified, as was national legislation in each of the selected African countries to denote the relevant avenues of protection and the mechanisms of protection which operate at each level.

The current members of the African Regional Intellectual Property Organisation (ARIPO) include Botswana, The Gambia, Ghana, Kenya, Malawi, Rwanda, the United Republic of Tanzania, Uganda, and Zimbabwe (African Regional Intellectual Property Organisation, 2023). South Africa and Nigeria, while not members under ARIPO, have observer status (Harakenzo World Patent and Trademark, 2023). Under the Harare Protocol, ARIPO can grant and register patents, industrial designs and utility models on behalf of contracting countries. The Protocol is currently in force in 18 of the 19 member countries (the exception being Somalia).

All of the countries under study have enacted patent and copyright statutes which are similar in many ways. The legislation is captured in Supplementary Annex I and Supplementary Table S5. All countries offer copyright protection (and share similar provisions) for the protection of computer programs and compilations of data and/or data tables. Any parties seeking protection for their data records and computer programs can obtain them in all 12 African countries.

Patent protection is available in all selected African countries for AI applications such as core inventions relating to novel advances in model architectures or to the techniques themselves. Other patentable innovations include: novel ways of generating a training set or model; trained models (the most common being AI as a tool to solve a particular problem); and smart AI-enhanced products and health monitoring devices.

#### Relevant authorities

All jurisdictions have yet to establish authorities or oversight mechanisms mandated to regulate AI. However, regulatory bodies and authorities overseeing data protection, ICTs, and medical devices will play a role in the regulation of AI systems and application in healthcare. The establishment of such authorities is set out in Supplementary Annex I at Supplementary Table S6.

Three of the twelve countries have established relevant committees to guide the uptake of emerging technologies, each of which has produced 4IR strategy documents. In 2018, the Kenyan Cabinet Secretary for ICT appointed the *ad hoc* Distributed Ledger and Artificial Intelligence Taskforce to: critically review AI, contextualise how its application could achieve the goals of, *inter alia*, universal healthcare and enhanced government service provision and to ‘prepare an implementation strategy with key performance indicators and clear delivery timelines’ (Authority of the Republic of Kenya, 2018). Similarly, in 2018, Uganda established the National Expert Taskforce on the 4IR, which was aimed at determining the state of 4IR technologies in the country, reviewing the legal and policy landscape, recommending a 4IR strategy and national institutional framework, and advising on a national framework intended to solidify the country as a 4IR regional hub (Ministry of ICT & National Guidance, 2022). In South Africa, the Presidential Commission on the 4IR (PC4IR) was mandated (South African Government 1A, 2022) to develop an integrated national strategy and advise on the advancement of global competitiveness, research and skills development. The PC4IR is also tasked with making recommendations to clearly articulate the roles of the state, constitutional actors, and citizens (South African Government 1B, 2022).

In addition, Rwanda and South Africa have established Centres for the Fourth Industrial Revolution–multi-stakeholder initiatives intended to focus on data governance, AI and machine learning (World Economic Forum, n.d.; Centre for the Fourth Industrial Revolution South Africa, 2022). These remain the only African countries that have partnered with the World Economic Forum in developing a network ‘connecting technology policy experts and stakeholders across 16 advanced and emerging economies’ (World Economic Forum, n.d.).

## Conclusion

This work demonstrates that in the 12 selected African countries, AI in healthcare, including in health research, is regulated. However, a diverse and fragmented progress indicates that significant work is yet to be done. Certain selected African countries have made limited progress and all of the 12 selected African countries are at an early stage in their AI regulatory journey. Notwithstanding regulatory developments, where found, development is often either of general application to all technology or adapted from other older digital technology types.

Encouragingly, certain sectors that inform AI development such as data protection have seen increased development in recent years. This is to be welcomed as exchanging and sharing knowledge, data, and efficiencies between African countries is transformative and can help to build common AI capacity across Africa. This is of particular importance in health research. We have identified the AI-relevant regulators and regulations–and instances where regulatory bodies and regulation are either absent or require strengthening. What is now required is a concerted effort by those regulators to engage with each other, and with health sector stakeholders and health researchers, to address gaps and deficiencies through domestic legal reform and policy development.

Importantly, where a regulatory framework exists, its role, we suggest, should be two-fold: to both prevent AI-related harm and to promote AI innovation across Africa. However, whether extant regulation achieves this and is suitable in the selected target countries for the purposes of AI adoption remains unclear. Where digital health policies and professional guidelines are absent or inadequate they need to be adopted or amended to enable responsible development and deployment of AI both in face-to-face patient care and telemedicine solutions, without stifling innovation. On AI innovation, AI generative tools promise to produce value. However, questions arise about whether these products qualify for intellectual property rights given that there is argument over whether they are created by a human or AI. African countries can certainly benefit by providing guidance on this important matter. In addition, there is limited African scholarship on AI ethics and policy, which makes for important and necessary future research in Africa.

Accordingly, Africa stands to gain from the proliferation of international and sector-specific ethical standards, guidelines, and policies, developed in a response to create “trustworthy,” “transparent,” and “responsible” AI (European Commission, 2019; Jobin et al., 2019; OECD Expert Group on AI, 2019). While certain jurisdictions outside of the African continent have proposed specific AI legislation, most notably the proposed European Union “Regulation laying down harmonization rules on Artificial Intelligence” (the “EU AI Act”) (European Commission, 2021) and the US Algorithmic Accountability Act of 2022, other regions have opted for alternative approaches to AI regulation such as those under consideration in the United Kingdom White Paper on AI regulation published in March 2023 (UK, 2023). In the Global South, the Brazilian Artificial Intelligence Bill, enacted in 2021, contains principles, rights, and duties for the use of artificial intelligence in Brazil, Uruguay adopted an AI strategy on responsible AI in public administration in 2019, Peru and Colombia issued National AI Strategies, and Chile, a National AI Policy.

Africa can certainly draw on these perspectives and benefit from more general and broader policy guidelines and regulation on AI, and specifically on AI in healthcare and health research. The African Union too can play a role in directing such initiatives. The post-colonial reach of digitised data and AI create challenges to Africa’s quest for digital sovereignty. However, Africa and indeed most of its nation states have been slow to agree on key digital and data governance measures. For example, as the uptake of the African Union Convention on Cyber Security and Personal Data Protection has demonstrated, progress is often both long and slow (African Union, 2014; Gwagwa and Townsend, 2023). What an appropriate and effective approach for AI regulation would look like for Africa and its individual sovereign nation states and how it may be implemented is an area for urgent and much needed future research.

We identify the role of the local community and African society in establishing principles and in participation and engagement in regulatory policy-making. The AI ecosystem is global, necessitating greater international collaboration and agreement of standards, frameworks, and guidance. Thus, the need exists to align the African position with international standards. However, while the Global North can inform African regulatory development and work at a global level to implement effective AI standards for safety, for example, and can bind countries to certain rules (Metzinger, 2022), we caution against a position where the normative principles and values that guide global AI adoption do not integrate as many perspectives as possible, including African viewpoints. Consideration must be had of the many unique historical and current challenges presenting in Africa. As suggested in Goffi and Momcilovic, we endorse an approach that embraces multiculturalism, and which offers due respect for cultural diversity in AI governance. An approach that is respectful of a variety of ethical perspectives and which involves multilateral debates at local and global levels (Goofy and Momcilovic, 2022).

Notwithstanding the emerging global approaches, we recommend that AI regulation in Africa is best served by being pro-innovation while addressing the many AI practices that carry unacceptable or high risk to health, safety, and human rights infringements. A framework for AI regulation in Africa, we suggest, should follow a cautious, yet proactive and balanced regulatory approach–one that is risk-based, rights-preserving, agile, adaptive, and innovation-supportive. In addition, we suggest that an effective African governance approach should include various governance tools–a combination of hard and soft law-including: 1) mechanisms to capture AI due diligence; 2) principles of transparency, explainability, and accountability; 3) be human-centric; and 4) make provision for AI auditing, assessment, and review. We recommend that an African approach be both risk-based and rights-based. This is premised on the understanding that AI systems have certain characteristics (*inter alia*, an opacity, complexity, dependency on data, and a capacity for autonomous behaviour) that can adversely and significantly affect fundamental human rights–rights to data privacy, transparency and disclosure, autonomy and self-determination, and the like.

Regulators in Africa have an increasing responsibility to address the immediate and significant concerns of algorithmic bias and fairness in the adoption of AI in Africa. AI stands, not only to potentially produce biased outcomes, but also to amplify and perpetuate patterns of general systemic and structural social bias, such as race- and gender-discrimination (Susskind, 2018; Kearns and Roth, 2020). Algorithmic injustice arises when patterns of marginalisation, imprinted in the historical data that shape the training and the testing of the system, produce individual predictive anomalies that, if left unchecked, inform a pernicious feedback loop of further exacerbating future down-stream systemic and structural injustice within larger groupings (Kearns and Roth, 2020; Glickman and Sharot, 2022). Algorithmic injustice is aggravated where data are under-representative or exclude certain categories of persons resulting in the exacerbation of long-standing societal biases that exist in relation to protected features like race and gender, and are magnified by virtue of their reach and scale.

Better or worse futures in the region will be determined, we suggest, in large part by clearly understanding and articulating the perspectives of previously marginalized and silenced voices and allowing them to be part of the AI conversation. Zimmermann et al. argue that “algorithmic injustice is not only a technical problem, but also a moral and political one, and that addressing it requires deliberation by all of us as democratic citizens.” Accordingly, accountability for addressing these injustices becomes shared, rather than that only “offloaded and outsourced to tech developers and private corporations” (Zimmermann et al., 2020).

The overarching idea too is that the higher the risk level, the greater the need for obligations to be placed on the AI system (and those developing and deploying it) and for human protection. Due regard should also be given to those activities that should be prohibited or otherwise curtailed, for example, amongst others, those outlined in the EU AI Act, that is, the use of systems that manipulate human behaviour and/or exploit persons’ vulnerabilities and social scoring systems. While AI systems pose many immediate risks to Use short dashes in order to be consistent with the rest of the paper also pose broader, longer-term social harms and large-scale, highly consequential risks that are often difficult to predict ex ante (Kolt, 2023). Further research and focus should be placed on these longer-term risks and on those that have broader social impact in a proposed African AI regulatory solution.

## Data Availability

The original contributions presented in the study are included in the article/Supplementary Material, and further inquiries can be directed to the corresponding author.
