# Supplementary material for: Mapping the regulatory landscape of AI in healthcare in Africa
Source: Front Pharmacol. 2023 Aug 24;14:1214422. doi: 10.3389/fphar.2023.1214422 (PMC10484713; doi:10.3389/fphar.2023.1214422)
Supplement: Supplementary file 1 [file Table1.docx]

**Annex I**

**Table 1: National digital health policies responsible for guiding the implementation of e-health/m-health/telemedicine initiatives in each jurisdiction.**

| **Country** | **Digital Health/e-Health/m-Health/Telemedicine Policy** |
| --- | --- |
| Botswana | eHealth Strategy of Botswana 2020-2024 |
| Cameroon | National Digital Health Strategic Plan 2020-2024 |
| The Gambia | Part of broader health policy – The National Health Policy 2021-2030 |
| Ghana | National E-Health Strategy 2010 |
| Kenya | Kenya National eHealth Policy 2016-2030  Kenya has an E-health Bill of 2021 |
| Malawi | National Digital Health Strategy 2020-2025 |
| Nigeria | National Health ICT Strategic Framework 2015-2020 |
| Rwanda | Part of broader health policy – The Fourth Health Sector Strategic Plan 2018-2024 |
| South Africa | National Digital Health Strategy for South Africa 2019-2024 |
| Tanzania | National Digital Health Strategy 2019-2024 |
| Uganda | Uganda National eHealth Strategy 2017-2021 |
| Zimbabwe | Zimbabwe e-health strategy 2012-2017 |

**Table 2: Health profession and health research legislation aimed at governing the medical profession, medical research activities and medical devices registered in each jurisdiction.**

| **Country** | **Statute regulating health profession** | **Regulation of medical research** | **Statute regulating of AI SaMD** | **Medical device regulatory authority** |
| --- | --- | --- | --- | --- |
| Botswana | Botswana Health Professions Act, CAP 61:02 | Guidelines for regulating the conduct of clinical trials using medicines in human participants. | Medicines and Related Substances Act of 2013 | Botswana Medicines Regulatory Authority |
| Cameroon | Law relating to the Organisation and Practice of Medicine Law No. 90-036 of 10 August 1990 | Medical research in Cameroon is regulated by Law No. 2022/008 of 27 April 2022 relating to medical research involving human subjects in Cameroon. | Public Health Act, Act 851 of 2012 | Food and Drug Administration |
| The Gambia | Public Health Act of 1989 | Guidelines for Clinical Trials in Humans | Medicines and Related Products Act, 2014 | Medicines Control Agency |
| Ghana | Medical and Dental Act, 1972 N.R.C.D. 91. | Guidelines for Clinical Trials | Public Health Act 851, 2012 | Food and Drugs Authority |
| Kenya | Medical Practitioners and Dentists Act 20 of 1977 | Pharmacy and Poison Amendment Act, 2021 | Pharmacy and Poison Amendment Act, 2021 | Pharmacy and Poisons Board (PPB) |
| Malawi | Medical Practitioners and Dentists Act No. 17 of 1987 | National Health Sciences Research Committee | Pharmacy and Medicines Regulatory Act 9 of 2019 | Pharmacy and Medicines Regulatory Authority |
| Nigeria | Medical and Dental Practitioners Act 2004 | National Health Act No. 8 of 2014  Public Health Act No. 851 of 2012 | National Agency for Food and Drug Administration and Control ACT Cap N.1 LFN 2004 | National Agency for Food and Drugs Administration and Control (NAFDAC) |
| Rwanda | Registration and Licensing Policy | Guideline on Inspecting clinical trials in Rwanda | Law Nº 003/2018 | Rwanda Food and Drug Administration |
| South Africa | The Health Profession Act 56 of 1964 | South African Medical Research Council Act 58 of 1991 | Medicines and Related Substances Act 101 of 1965 | South African Health Products Regulatory Authority |
| Tanzania | The Medical Council of Tanganyika (Exams and Registration Procedures) Regulations, 2018 | National Institute for Medical Research, Act No. 23 of 1978 | Tanzania Medicines and Medical Devices Act, Cap 219 of 2019 | Tanzania Medicines and Medical Devices Authority |
| Uganda | Medical and Dental Practitioner Act 1998 | Uganda National Health Research Organisation Act | No | National Drug Authority |
| Zimbabwe | Health Professions Act | Health Research Act |  | Medicines Control Authority of Zimbabwe |

**Table 3: Data protection legislation and the level of means of governance provided in each jurisdiction.**

| **Country** | **Data Protection Act/Bill** | **Coverage** | **Information Regulator/Commissioner** |  |
| --- | --- | --- | --- | --- |
| Botswana | [Data Protection Act, 2018](https://www.bocra.org.bw/sites/default/files/documents/DataProtectionAct.pdf) | Full | Yes |  |
| Cameroon | No specific law, although there is partial coverage through constitutional rights and sectoral regulation of consumer and ICT law | Partial | – |  |
| The Gambia | No specific law. The Data Protection and Privacy Policy, 2019, sets the framework for data protection. Draft Data Protection and Privacy Bill. | Partial | – |  |
| Ghana | [Data Protection Act, 2012](https://nita.gov.gh/wp-content/uploads/2017/12/Data-Protection-Act-2012-Act-843.pdf) | Full | Yes |  |
| Kenya | Data Protection Act [No. 24 of 2019](http://kenyalaw.org:8181/exist/kenyalex/actview.xql?actid=No.%2024%20of%202019) | Full | Yes |  |
| Malawi | Data Protection Bill, 2021 | Partial | – |  |
| Nigeria | Data Protection Regulation, 2019  Data Protection Bill, 2020. | Full | Yes |  |
| Rwanda | Law Nº 058/2021 of 13/10/2021 relating to the protection of personal data and privacy. | Full | Yes |  |
| South Africa | [Protection of Personal Information Act 4 of 2013 \| South African Government](https://www.gov.za/documents/protection-personal-information-act) | Full | Yes |  |
| Tanzania | Personal Data Protection Act of 2022. | Full | Yes |  |
| Uganda | [Data Protection and Privacy Act, 2019](https://www.dataguidance.com/legal-research/data-protection-and-privacy-act-2019)  The Data Protection and Privacy Regulations 2021. | Full | Yes |  |
| Zimbabwe | Data Protection Act No. 5 of 2021  [65384-T Cyber & Data Protection Act](https://www.veritaszim.net/sites/veritas_d/files/Data%20Protection%20Act%205%20of%202021.pdf). | Full | Yes |  |

**Table 4: Consumer protection, ECT and cybercrime legislation responsible for governing relevant online and commercial activities in each jurisdiction.**

| **Country** | **Consumer protection law** | **Electronic communication and transactions law** | **Cybercrime law** |
| --- | --- | --- | --- |
| Botswana | Consumer Protection Act, 2018 – strict product liability provided for in section 18. | Electronic Communications and Transactions Act, 2014 | Cybercrime and Computer Related Crimes Act, 2018 |
| Cameroon | Law No. 2011/012 of 06 May 2011 on Consumer Protection in Cameroon | Decree No. 2013/0399/PM of 27 February 2013 relating to e-communications consumer protection | Law N° 2010/012 of 21 December 2010 Relating to Cyber security and Cyber criminality in Cameroon |
| The Gambia | The Gambia Consumer Protection Act, 2014 – strict product liability provided for in Part V. | None | None |
| Ghana | Sale of Goods Act, 1962 – strict product liability provided for in Part VI. | Electronic Communications Act, 2008  Electronic Transactions Act, 2008 | Cybersecurity Act of 2020 |
| Kenya | Consumer Protection Act, 2012 – strict product liability provided for in section 16 of Sale of Goods Act 19 of 1964 read with section 55 of Consumer Protection Act. | None | The Computer Misuse and Cybercrimes Act, 2018 |
| Malawi | Consumer Protection Act 14 of 2003 – strict product liability provided for in sections 33, 41 & 52. | Electronic Transactions and Cyber Security Act, 2017 | Electronic Transactions and Cyber Security Act, 2017 |
| Nigeria | Federal Competition and Consumer Protection Act, 2018 – strict product liability provided for in section 136. | None | Cybercrime Act, 2015 |
| Rwanda | Law Nº 36/2012 of 21/09/2012 Relating to Competition and Consumer Protection – strict product liability provided for in article 49. | Law Nº 18/2010 of 12/05/2010 Relating to Electronic Messages, Electronic Signatures and Electronic Transactions | Law Nº 60/2018 of 22/8/2018 on Prevention and Punishment of Cyber Crimes |
| South Africa | Consumer Protection Act 68 of 2008 – strict product liability provided for in section 61. | Electronic Communications and Transactions Act 25 of 2002 | Cybercrimes Act 19 of 2020 |
| Tanzania | The Fair Competition Act 8 of 2003 – strict product liability provided for in sections 44 & 47. | The Electronic Transactions Act, 2015 | The Cybercrime Act, 2015 |
| Uganda | Sale of Goods and Supply of Services Act 10 of 2018 – strict product liability provided for in Part V. | Electronic Transactions Act 8 of 2011 | The Computer Misuse Act 2 of 2011 |
| Zimbabwe | Consumer Protection Act 5 of 2019 – strict product liability provided for in section 16. | None | Cybersecurity and Data Protection Bill, 2019 |

**Table 5: National intellectual property legislation in each jurisdiction and country membership of relevant regional organisations.**

| **Country** | **Patent** | **Copyright** | **ARIPO** | **OAPI** | **Coverage** |
| --- | --- | --- | --- | --- | --- |
| Botswana | Industrial Property Act, 2010 | The Copyright and Neighbouring Rights Act 8 of 2000 | X |  | Full |
| Cameroon | The Bangui Agreement of 1977 as amended | Law No 2000/11 of 19 December 2000 on Copyright and Neighbouring rights |  | X | Partial |
| The Gambia | The industrial Property Act (CAP 95:03) of 2010 | Copyright Act 10 of 2004 | X |  | Full |
| Ghana | Patents Act 657 of 2003 | Copyright Act 690 of 2005 | X |  | Full |
| Kenya | Industrial Property Act 3 of 2001 | Copyright Act 12 of 2001 | X |  | Full |
| Malawi | Patents Act (49:02) | Copyright Act 26 of 2016 | X |  | Full |
| Nigeria | Patents and Designs Act 1971 | Copyright Act 1988 | Observer status |  | Full |
| Rwanda | The Law N° 31/2009 of 26/10/2009 on the Protection of Intellectual Property | The Law N° 31/2009 of 26/10/2009 on the Protection of Intellectual Property | X |  | Full |
| South Africa | The South African Patents Act 57 of 1978 | The South African Copyright Act 98 of 1978 | Observer status |  | Full |
| Tanzania | The Patents (Registration) Act, Cap. 217 (“Patents Act”) | Copyright and Neighbouring Rights Act 7 of 1999 (Copyright Act) | X |  | Full |
| Uganda | The Patents Act of 1993 | The Copyright and Neighbouring Rights Act 2006 | X |  | Full |
| Zimbabwe | The Patents Act | Copyright and Neighbouring Rights Act | X |  | Full |

**Table 6: Regulatory bodies influential in the oversight and use of AI medical technologies in each jurisdiction.**

| **Country** | **Data protection** | **E-health** | **ICT** | **Medical devices** |
| --- | --- | --- | --- | --- |
| Cameroon | None | Ministry of Public Health | Telecommunications Regulatory Board | Food and Drugs Authority |
| Botswana | The Information and Data Protection Commission | Ministry of Health and Wellness | Botswana Communications Regulatory Authority | Botswana Medicines Regulatory Authority |
| The Gambia | None | Ministry of Health | The Gambia Information and Communication Technology Agency | Medicines Control Agency |
| Ghana | Data Protection Commission | Ministry of Health | National Information Technology Agency | Food and Drugs Authority |
| Kenya | Office of the Data Protection Commissioner | Ministry of Health | Communications Authority of Kenya | Pharmacy and Poisons Board |
| Malawi | None | Ministry of Health | Malawi Communications Regulatory Authority | Pharmacy and Medicines Regulatory Authority |
| Nigeria | Nigeria Data Protection Bureau | Federal Ministry of Health | Nigerian Communications Commission | National Agency for Food and Drug Administration and Control |
| Rwanda | National Cyber Security Authority | Ministry of Health | Rwanda Utilities Regulatory Authority | Rwanda Food and Drugs Authority |
| South Africa | The Information Regulator | National Department of Health | Independent Communications Authority of South Africa | South African Health Products Regulatory Authority |
| Tanzania | None | Ministry of Health | Tanzania Communications Regulatory Authority | Tanzania Medicines and Medical Devices Authority |
| Uganda | Personal Data Protection Office | Ministry of Health | Uganda Communications Commission | National Drug Authority |
| Zimbabwe | The Postal and Telecommunications Regulatory Authority of Zimbabwe | Ministry of Health and Child Care | The Postal and Telecommunications Regulatory Authority of Zimbabwe | Medicines Control Authority of Zimbabwe |
