# Supplementary material for: Mapping the regulatory landscape of AI in healthcare in Africa
Source: Front Pharmacol. 2023 Aug 24;14:1214422. doi: 10.3389/fphar.2023.1214422 (PMC10484713; doi:10.3389/fphar.2023.1214422)
Supplement: Supplementary file 2 [file Table2.docx]

**Annex II**

Table 1: Keywords used

| **AI and Data Protection** | · “Algorithm”  · “Algorithmic bias”  · “Algorithmic injustice”  · “Artificial intelligence”  · “Automated decisions”  · “Automated decision making”  · “Data protection”  · “Design principle[s]”  · “Disruptive technology”  · “Disruptive technologies”  · “E-health”  · “Ehealth”  · “Electronic health”  · “Emerging technology”  · “Emerging technologies”  · “Ethics by design”  · “Ethics for design”  · “Ethics in design”  · “Fourth industrial revolution”  · “Healthcare”  · “Health data”  · “Personal information”  · “Privacy”  · “Privacy by default”  · “Privacy by design”  · “Privacy for design”  · “Privacy in design”  · “Sensitive data”  · “Software”  · “Special personal information”  · “Technical design”  · “Technical principle[s]”  · “Telemedicine”  · “Value design”  · “Value sensitive design” |
| --- | --- |
| **Constitutional Provisions, Consumer Protection & ICT Regulation** | **Constitutional provisions:**  · “Academic freedom”  · “Data protection”  · “Freedom of expression”  · “Healthcare”  · “Innovation”  · “Privacy”  · “Right to data protection”  · “Right to healthcare”  · “Right to privacy”  · “Scientific freedom”  · “Scientific innovation”  **Consumer protection:**  · “Algorithm”  · “Consumer”  · “Consumer protection”  · “Consumer rights”  · “Goods”  · “Hardware”  · “Product liability”  · “Software”  · “Software as goods”  · “Source”  · “Source code”  · “Strict liability”  · “Technology”/”Technologies”  **ICT/E-legislation:**  · “Censorship”  · “Content control”  · “Cyber-crimes”  · “Cyber law”  · “Cyber security”  · “Electronic communications”  · “Electronic transactions”  · “Fact checking”  · “Information and communications technology”  · “Internet service provider”  · “Limitations on liability”  · “Limited liability”  · “Misinformation”  · “Service provider”  · “Service provider liability” |
| **Digital Health/e-Health & Medical Device Regulation** | · “Consent”  · “Health care practitioner”  · “Health care professional”  · “Personal information”  · “Privacy”  · “Registration”  · “Right to health”  · “Telemedicine” |
| **Intellectual Property** | · “AI”  · “Artificial intelligence”  · “Collation”  · “Collection”  · “Computer”  · “Copyright”  · “Data”  · “Database”  · “Dataset”  · “Exclusions”  · “Exclusive”  · “Genetic data”  · “Genomic data”  · “Health”  · “Health data”  · “Information”  · “Infringement”  · “Innovation”  · “Intellectual property”  · “IP”  · “License”  · “Open”  · “Open innovation”  · “Open science”  · “Ownership”  · “Patent”  · “Point”  · “Programme”  · “Protection”  · “Research”  · “Trade secret” |

Table 2: Databases searched

| All countries | 1. African Union 2. East African Community 3. Economic Community of West African States 4. Intergovernmental Authority on Development 5. South African Development Community 6. The Common Market for Eastern and Southern Africa 7. Afriwise 8. World Intellectual Property Organization 9. Ellipsis 10. DataGuidance 11. P4H 12. African Regional Intellectual Property Organisation 13. African Legal Information Institute 14. World Legal Information Institute 15. The World Law Guide (Lexadin) 16. LexisNexis 17. Internet Governance Forum 18. Jutastat 19. ICT Policy Africa 20. ICTworks 21. ARAZY Group 22. World Health Organisation Office for Africa |
| --- | --- |
| Botswana | AI & Data Protection   1. SmartBots 2. Vision 2036 3. Botswana’s Digital and Innovation Hub 4. Blackhall Publishing Laws of Botswana 5. Botswanalii 6. Botswana Innovation Hub 7. Botswana Institute for Development Policy Analysis 8. Botswana Institute for Technology, Research and Innovation 9. Government of Botswana 10. Ministry of Health & Wellness, Botswana   Digital health/e-Health and Medical device regulation   1. Botswana Government Gazette 2. Ministry of Health and Wellness 3. Laws of Botswana 4. Botswana Medicines Regulatory Authority (BoMRA)   Consumer Protection   1. Competition and Consumer Authority |
| Cameroon | AI & Data Protection   1. Agency of Regulation of Telecommunications 2. Cameroon Government Certification Authority 3. National Agency for Information and Communication Technologies 4. Presidency of the Republic of Cameroon 5. Cyrilla 6. Fratel 7. GovInfo   Digital Health/e-Health and Medical devices   1. Food and Drugs Authority of Cameroon 2. Ministry of Public Health 3. Medical Council-MEDCAMER-Cameroon Medical Doctors   Consumer Protection   1. Telecommunications Regulatory Board |
| The Gambia | AI & Data Protection   1. Gambia Competition and Consumer Protection Commission 2. Ministry of Information and Communications Infrastructure 3. Gnews for Gambia   Digital health/e-Health & Medical device regulation   1. Ministry of Health 2. Ministry of Information and Communications Infrastructure 3. Medicines Control Agency 4. The Medical and Dental Council of The Gambia   Intellectual Property   1. National Centre of Arts and Culture   Consumer Protection   1. The Gambia Competition and Consumer Protection Commission |
| Ghana | AI & Data Protection   1. Council for Scientific and Industrial Research 2. Data Protection Commission 3. Food and Drugs Authority 4. Ghana.GOV 5. Ghana Legal Information Institute 6. Institute for Scientific & Technological Information 7. Ministry of Communications and Digitalisation 8. Ministry of Environment, Science, Technology and Innovation 9. Ministry of Health 10. Parliament of Ghana 11. Parliament of Ghana Library Repository 12. Science and Technology Policy Research Institute   Digital Health/e-Health & Medical Device Regulation   1. Ministry of Health 2. Food and Drugs Authority of Ghana 3. Medical and Dental Council |
| Kenya | AI & Data Protection   1. Bioethics Society of Kenya 2. Kenya Law 3. Kenya Medical Research Institute 4. Kenya National Innovation Agency 5. Ministry of Health 6. Ministry of ICT, Innovation and Youth Affairs 7. National Commission for Science, Technology and Innovation 8. National Research Fund 9. Office of the Attorney General and Department of Justice 10. Office of the Data Protection Commissioner 11. Parliament of Kenya 12. The Kenya Institute for Public Policy Research and Analysis   Digital Health/e-Health & Medical Device Regulation   1. Pharmacy and Poisons Board 2. Ministry of Health 3. Kenya Medical Research Institute 4. Kenya Medical Practitioners and Dentist Council   Intellectual Property   1. Academic Kenya e-Repository 2. Kenya Law   Consumer Protection   1. Competition Authority of Kenya |
| Malawi | AI & Data Protection   1. Ministry of Information and Communications Infrastructure 2. Malawi Communications Regulatory Authority 3. Malawi Ministry of Information 4. Digital Malawi   Digital Health/e-Health & Medical Device Regulation   1. Ministry of Health 2. Medical Council of Malawi 3. Ministry of Education, Science and Technology 4. National Commission for Science and Technology 5. Pharmacy and Medicines Regulatory Authority   Consumer Protection   1. Competition and Fair-Trading Commission of Malawi |
| Nigeria | AI & Data Protection   1. National Information Technology and Development Agency 2. National Centre for Artificial Intelligence and Robotics 3. Federal Ministry of Information and Culture 4. Nigerian Communications Commission 5. Federal Ministry of Health   Digital Health/e-Health & Medical Device Regulation   1. Federal Ministry of Health 2. Medical and Dental Council of Nigeria 3. Federal Ministry of Information and Culture 4. Nigerian Institute of Medical Research 5. National Agency for Food and Drugs Administration and Control   Intellectual Property   1. The Trademarks, Patents and Designs Registry, Commercial Law Department, Federal Ministry of Industry, Trade and Investment 2. Federal Ministry of Industry, Trade and Investment   Consumer Protection   1. Federal Competition and Consumer Protection Commission |
| Rwanda | AI & Data Protection   1. Ministry of Information Communication Technology and Innovation 2. National Council for Science and Technology 3. Centre for the Fourth Industrial Revolution 4. Ministry of Health   Digital Health/e-Health   1. Ministry of Health 2. Rwanda Food and Drug Administration 3. Rwanda Medical and Dental Council 4. Rwanda Biomedical Centre 5. Rwanda National Health Research Registry 6. Rwanda Trade   Intellectual Property   1. Rwanda Development Board   Consumer Protection   1. Rwanda Inspectorate, Competition and Consumer Protection Authority |
| South Africa | AI & Data Protection   1. Academy of Science of South Africa 2. Council for Scientific and Industrial Research 3. Department of Health 4. Department of Science and Innovation 5. Health Professions Council of South Africa 6. Human Sciences Research Council 7. Information Regulator (South Africa) 8. National Advisory Council on Innovation 9. Protection of Personal Information Act 10. Public Health Institute of South Africa 11. South African Medical Research Council 12. Technology Innovation Agency   Digital Health/e-Health & Medical Device Regulation   1. Government Gazette of South Africa 2. Health Profession Council 3. South African Health Products Regulatory Authority 4. South African Medical Research Council 5. Department of Health South Africa   Intellectual Property   1. Department of Science and Technology 2. National Advisory Council on Innovation 3. South African Government 4. Southern African Legal Information Institute (SAFLII) 5. Technology Innovation Agency   Consumer Protection   1. National Consumer Commission |
| Tanzania | AI & Data Protection   1. Parliament of Tanzania 2. Government of Tanzania 3. Tanzania Academy of Sciences 4. Ministry of Information, Communication and Information Technology 5. Information and Communication Technology Commission 6. Ministry of Health 7. Ministry of Agriculture 8. Tanzania Commission for Science and Technology 9. Tanzania Communications Regulatory Authority 10. National Institute for Medical Research   Digital Health/e-Health & Medical Device Regulation   1. Ministry of Health 2. National Institute for Medical Research 3. Tanzania Medicines and Medical Devices Authority 4. The Medical Council of Tanganyika 5. Tanzania Investment Centre   Intellectual Property   1. Tanzania Commission for Science and Technology 2. Parliament of Tanzania 3. Government of Tanzania 4. Tanzania Legal Information Institute (Tanzlii)   Consumer Protection   1. Fair Competition Commission |
| Uganda | AI & Data Protection   1. Uganda Law 2. Uganda Medical Research Institute 3. Uganda National Health Research Organisation 4. Ministry of Health 5. Ministry of ICT, Innovation and Youth Affairs 6. National Commission for Science, Technology and Innovation 7. National Research Fund 8. Office of the Attorney General and Department of Justice 9. Office of the Data Protection Commissioner 10. Parliament of Uganda   Digital Health/e-Health & Medical Device Regulation   1. Ministry of Health 2. National Drug Authority 3. Uganda National Health Research Organisation 4. Uganda Medical Research Institute 5. Uganda Medical and Dental Practitioners Council   Intellectual Property   1. Uganda Law 2. Open Access Digital Repository   Consumer Protection   1. Uganda Communications Commission |
| Zimbabwe | AI & Data Protection   1. Zimbabwe Government Gazette 2. Zimbabwe Legal Information Institute 3. Medical and Dental Practitioners Council 4. Ministry of Health and Child Care 5. Government of Zimbabwe 6. Ministry of ICT, Postal and Courier Services 7. Ministry of Higher and Tertiary Education, Innovation, Science and Technology Development 8. Zimbabwe Centre For High Performance Computing   Digital Health/e-Health & Medical Device Regulation   1. Ministry of Health and Child Care 2. Medicines Control Authority of Zimbabwe 3. Research Council of Zimbabwe 4. National Biotechnology Authority 5. Zimbabwe Legal Information Institute   Intellectual Property   1. Ministry of Justice, Legal and Parliamentary Affairs 2. Department of Deeds, Companies, and Intellectual Property 3. Zimbabwe Legal Information Institute 4. The Reserve Bank of Zimbabwe   Consumer Protection   1. Consumer Council of Zimbabwe |
